# Regulation of Growth and Main Health-Promoting Compounds of Chinese Kale Baby-Leaf by UV-A and FR Light

**DOI:** 10.3389/fpls.2021.799376

**Published:** 2021-12-17

**Authors:** Rui He, Yamin Li, Shuying Ou, Meifang Gao, Yiting Zhang, Shiwei Song, Houcheng Liu

**Affiliations:** College of Horticulture, South China Agricultural University, Guangzhou, China

**Keywords:** UV-A, far-red light, *Brassica alboglabra*, glucosinolate, metabolism

## Abstract

Chinese kale baby leaves were hydroponically cultured under the basal light (Red: white LEDs = 2:3 at PPFD of 250 μmol·m^−2^·s^−1^) with different supplemental lighting, including individual ultraviolet-A (UV-A, 380 ± 10 nm, 20 μmol·m^−2^·s^−1^), far-red (FR, 735 ± 10 nm, 30 μmol·m^−2^·s^−1^) light, and their combination (UF) radiation in an artificial light plant factory. Effects of supplemental light qualities on morphology and physiology as well as health-promoting compounds of Chinese kale baby leaves were investigated. Application of UV-A and FR presented a positive effect on biomass, with a pronounced increase in petiole length, stem diameter, main stem length, and leaf area. Notably, plants under UF grew more vigorously than under other treatments. Higher levels of FRAP, vitamin C, total phenolic, and flavonoid were observed in plants under UV-A, while no striking changes or a decreasing trend recorded under FR and UF. Moreover, UV-A enhanced the glucosinolates (GLs) accumulation in Chinese kale baby leaves by increasing the predominant GLs (glucoraphanin and glucobrassicin) contents. RT-qPCR results indicated that UV-A upregulated the gene expressions of transcription factors and core structure genes related to GLs biosynthesis. However, downregulated or unchanged gene expressions of GLs biosynthesis-related genes in Chinese kale baby leaves were observed in FR and UF. Therefore, UV-A was benefited for the production of functional substances, while FR was conducive to a significant increase in crop yield. The combination of UV-A and FR, as a balance between yield and production of secondary metabolite, provided a new perspective for the application of artificial light in horticultural crop production.

## Introduction

Light not only provides energy for plant photosynthetic, but it is also the most indispensable environmental signal to trigger an ensemble of signal-transducing photoreceptors that regulate plant growth, development, and metabolism (Karpiński et al., 2013). Plants respond to different light environment *via* multiple types of photoreceptors, including red and far-red light receptor phytochromes (PHYs), blue and UV-A light receptors cryptochromes (CRYs), phototropins (PHOTs), and Zeitlupe family proteins, as well as UV-B light receptor UVR8 (Galvão and Fankhauser, 2015). Once photoactivated, photoreceptors closely interact with presumably other photoreceptors or players, such as HY5, COP1, PIFs (Saijo et al., 2003), regulating plant morphology and a metabolite across an entire plant life cycle.

Light-emitting diode lights have been widely applied to horticultural cultivation to increase biomass production and nutritional quality, with numerous advantages over traditional light sources (Qian et al., 2016; Holopainen et al., 2018; Jung et al., 2021). Various focuses were given on photosynthesis, morphology, yield, and phytochemical accumulation of vegetables affected by red and blue light radiation due to that these spectral regions efficiently absorbed by chlorophyll. Ultraviolet-A (UV-A) light and far-red (FR) light, kinds of invisible lights, have created new opportunities to alter plant morphological and physiological properties, which depend on their wavelengths, intensities, and irradiation time (Fukuyama et al., 2017; Li et al., 2020a; Zhang et al., 2020; He et al., 2021).

Impacts of UV-A radiation on various aspects of vegetative and reproductive growth, as well as secondary metabolites accumulation, have been intensively investigated in recent years. Generally, conclusions that UV-A was beneficial for phytochemicals production, in particular of phenolics and flavonoids, have well been drawn (Neugart and Schreiner, 2018), but it is still limited about the effects of UV-A on plants growth. Stimulatory effects on biomass production and morphology were found under UV-A supplementation in tomato seedlings (Mariz-Ponte et al., 2018), kale (Lee et al., 2019), and lettuce (Chen et al., 2019). However, a 10 μmol·m^−2^·s^−1^ UV-A (380 nm) supplement exerted an inhibitory effect on fresh and dry weight of “Red butter” lettuce, whereas no significant difference in “Yanzhi” plants (He et al., 2021).

Far-red (FR) light plays crucial roles in a wide range of physiological responses in plants, including stem length and leaf elongation, leaf area expansion, leaf moving upward, biomass increases or decreases (Legendre and van Iersel, 2021). FR (740 nm, 30 μmol·m^−2^·s^−1^) supplementation increased plant biomass of two lettuce cultivars, yielding greater leaf length, leaf width, fresh and dry weight (He et al., 2021). Similarly, plant dry mass, fruit number per plant, and average fruit fresh weight in tomato were significantly increased by supplemental FR (Ji et al., 2020).

Chinese kale (*Brassica alboglabra* Bailey) is a widely consumed vegetable in China and Southeast Asia, which is a rich source of antioxidants and anticarcinogenic compounds, including vitamin C, glucosinolates (GLs), and phenolic compounds. Glucosinolates are sulfur and nitrogen-containing secondary metabolites in the *Brassicaceae* family, functioning as powerful antioxidants. The active hydrolysed by-products, isothiocyanates, which are degradation products of GLs breakdown by myrosinases, are beneficial to humans in preventing types of diseases, such as cancer, inflammation, and some degenerative diseases (Kapusta-Duch et al., 2012). UV-A radiation at different light intensities or wavelengths was in favor of GLs accumulation in mature plants of red-leaf and green-leaf pak choi (Tang et al., 2020), baby leaves of Chinese kale and pak choi (Li et al., 2020b), as well as broccoli sprouts (Gao et al., 2021), while FR seemed to be a negative factor in GLs accumulation in *Brassicaceae* vegetables (Steindal et al., 2016; Li et al., 2021). Supplemental FR was likely more efficient to increase biomass production by enlarging the leaf area and improving the ability of light interception, while it might result in lower phytochemicals contents in plants due to the shade avoidance syndrome (SAS) induced by FR light (Li et al., 2020a; He et al., 2021). Indeed, UV-A was beneficial for phytochemicals production, in particular of health-promoting compounds (Neugart and Schreiner, 2018). Although numerous studies have revealed the effects of UV-A or FR alone on vegetative growth, the interactive effect of UV-A and FR on vegetative growth and phytochemicals contents in plants is still limited. The combination of FR and UV-A might as a balance between higher yields and better quality in an artificial light plant factory. The objectives of this study were to quantify the effects of UV-A, FR, and combination of UV-A and FR on the growth and health-promoting phytochemicals in Chinese kale baby leaves.

## Materials and Methods

### Plant Material and Growth Conditions

This study was performed in an artificial light plant factory in South China Agricultural University. Three cultivation frames with 6 layers were equally divided into 4 individual experimental units. Shading clothes were placed around the experimental units in order to prevent light interference between treatments. The adjustable LED panels (150 × 30 cm; Chenghui Equipment Co., Ltd, Guangzhou, China) with red LEDs (660 ± 10 nm), white LEDs (peak at 440 nm), UV-A (380 ± 10 nm), and FR (735 ± 10 nm) LEDs were used as light sources, which mounted in each cultivation unit in, horizontally, 30 cm above the cultivation plate.

The Chinese kale (*Brassica alboglabra* Baile, “Lvbao,” from GLseed Co., Ltd, Guangzhou, China) seeds were germinated in a moist sponge block (2 × 2 × 2 cm) and cultured under white LED (300 μmol·m^−2^·s^−1^, 12/12 h) light after sprouting. Seedlings were fertilized with 1/4 strength Hogland nutrient solution. The composition of the full-strength nutrient solution was presented in Supplementary Table 1. Two weeks later, the seedlings with three expended true leaves were transplanted into a recirculating hydroponic culture system with density of 42 plants per plate (95 × 60 × 3 cm), and three plates containing 126 plants were subjected to each supplemental light treatment described below. The 1/2 strength of the nutrient solution (pH ≈ 6.5; EC ≈ 1.6 ms·cm^−1^) was recirculated automatically for 10 min every half hour.

### Supplemental Light Treatments

A combination of red and white LEDs (R:B = 2:3) with a light intensity of 250 μmol·m^−2^·s^−1^ was used as basal light (CK). The different light treatments were as follows: 20 μmol·m^−2^·s^−1^ UV-A light added to basal light (UV-A), 30 μmol·m^−2^·s^−1^ FR added to basal light (FR), and 20 μmol·m^−2^·s^−1^ UV-A and 30 μmol·m^−2^·s^−1^ FR added to basal light (UF). The photoperiod was 12 h per day in all treatments. The light spectral distribution was measured using a spectroradiometer (ALP-01, Asensetek, Taiwan) on the Chinese kale baby leave plants canopy level (Supplementary Figure 1).

### Morphological Measurements

Twelve baby-leaf plants per treatment were destructively determined at 3, 6, 9, 12 days after treatment initiation. Shoot (the whole plant without root) and root fresh weight were measured using an electronic balance. Subsequently, shoots and roots of plants were oven-dried separately to a constant mass at 70°C for shoot and root dry weight determination. Petiole length of the third true leaf on each plant and main stem length (length from cotyledons to point of growth) using a ruler, while the stem diameter at the widest point of each plant was measured using a digital caliper. The total leaf area was determined using ImageJ 1.42 software (https://imagej.nih.gov/ij).

**Figure 1:**
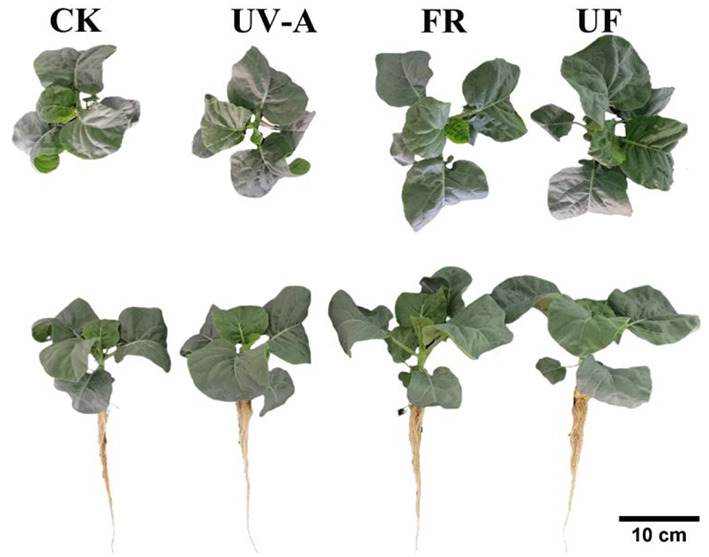
The Chinese kale baby-leaves morphology at 12 days after treatments.

The samples used for biochemical analysis and gene expression determinations were collected at 6th day (6 day) under treatments. Three biological repetitions were used in this study. Each biological repetition contained six plants. The shoots of baby-leave plants were immediately frozen in liquid nitrogen and stored at −80°C. The morphology of Chinese kale grown with 12 days of lighting treatments is shown in Figure 1.

### Phytochemicals Measurements

The ferric-reducing antioxidant power assay was according to the method decried by Benzie and Strain (Benzie and Strain, 1996). Frozen shoot tissue (about 0.5 g) was extracted with 8-ml 100% (v/v) ethanol for 30 min at 4°C in the dark, and then centrifuged at 3,000 rpm for 10 min at 4°C. The supernatants (0.4 ml) were mixed with 3.6-ml FRAP reagent [a solution containing a 0.3-M acetate buffer, 10-mM 2,4,6-tripyridyl-S-triazine (TPTZ), and 20-mM FeCl_3_ at a 10:1:1 ratio (v/v/v)]. Then, the mixture was maintained in a water bath at 37°C for 10 min. The absorbance was measured at 593 nm by UV spectrophotometry.

Total phenolic content was determined according to a Folin-Ciocalteu assay (Matsuura et al., 2013). Sample extracts were as a FRAP assay above. The 1-ml supernatant was mixed with 0.5-ml foline-phenol and 11.5-ml 26.7% (v/v) Na_2_CO_3_, 7-ml distilled water. After reacting at 26°C for 2 h, absorbance was read at 510 nm by a UV spectrophotometer.

The total flavonoid content was determined by the aluminum nitrate method (Xie et al., 2015). Briefly, 1-ml extract solution (extracted as FRAP) was added to 11.5 ml 30% ethanol and 0.7-ml 5% NaNO_2_. After 5 min, the reaction solution was mixed with 0.7-ml 10% Al (NO_3_)_3_, and 5-ml 5% NaOH was added after further 6 min. The absorbance was measured at 760 nm using the UV spectrophotometer after 10 min.

The vitamin C (VC) content was determined using the method reported by Kampfenkel et al. (1995). Frozen shoot tissue (0.5 g) was homogenized in 25-ml 5% oxalic acid. After filtering, 10-ml extract solution was mixed with 1-ml phosphate-acetic acid, 2-ml 5% vitriol, and 4-ml ammonium molybdate. The absorbance was recorded at 705 nm by the UV spectrophotometer.

### Minerals Measurements

The minerals measurement was performed according to Waterland (Waterland et al., 2017). Oven-dried samples power (0.2 g) was digested with 4-ml 70% HNO_3_ (v:v) for 4 h. The extract was filtered and adjusted to a total volume of 20 ml with deionized distilled water. Mineral concentrations were determined by inductively coupled plasma spectrometry (Optima 2100DV; Perkin Elmer Corp., Waltham, MA, USA).

### Glucosinolate Composition and Contents

Glucosinolate composition and contents were extracted, proposed by previously described by Li, with modifications (Li et al., 2021). Freeze-dried samples (200 mg) mixed with 4-ml 70% methanol were kept in water bath (80°C) for 20 min. The supernatant was collected after being centrifuged at 4,000 rpm for 10 min. The supernatant was transferred into columns filled with 500-μl DEAE-Sephadex A-25 (Sigma Chemical Co., Saint Louis, USA), which had been prewashed with 1-ml 6-M imidazole formate and a 0.02-M sodium acetate buffer, respectively. After incubating overnight with 100-μl 0.1% arylsulphatase (Sigma, St. Louis, MO, USA), desulphoglucosinolates were eluted with 2-ml distilled water. The total eluate filtered through a 0.22-μm membrane filter.

Desulphoglucosinolates were analyzed by HPLC; analyses were conducted on a Waters e2695 liquid chromatograph (Waters Crop., Milliford, MA, USA) with a reversed-phase C 18 column (5 μm, 250 × 4.6 mm; Waters SunFire C18, Waters, USA). The column was maintained at 30°C and 20-μl injection volume. A binary gradient was used: 0-32 min 0-20% eluent A; 32–38 min 20% A; 39–40 min 20–100% A; the eluents were (A) acetonitrile and (B) distilled water, with a flow rate of 1 ml·min^−1^. The detection wavelength was recorded at 229 nm. Sinigrin obtained from Sigma (St. Louis, MO, USA) was used as an internal standard for quantitation analysis, and the results were presented as μmol·g^−1^DW (dry weight) of Chinese kale baby leaves.

### RNA Isolation and qRT-PCR Analyses

Total RNA was extracted from 100 mg of frozen tissue using RNAex Pro Reagent (Accurate Biotechnology Co., Ltd., Hunan, China) for three biological repeats. RNA samples, whose OD260/280 and OD260/230 values were within 1.8–2.2, were selected to synthesize complementary DNA (cDNA) using Evo M-MLV RT for PCR Kit (Accurate Biotechnology Co., Ltd., Hunan, China).

Quantitative real time PCR (qRT-PCR) reactions were performed using LightCycler 480 Real-Time PCR system (Roche, Basel, Switzerland) with 10-μl Real Time system composed of 5-μl TB green (TaKaRa Bio, Inc., Dalian, China), a 1-μl cDNA sample, 3.2-μl ddH_2_O, a 0.4-μl forward primer, and a 0.4-μl reverse primer. The amplification program consisted of a 30-s initial step at 95°C, followed by 40 cycles of 5 s at 95°C and 30 s at 60°C. The primer sequences used are listed in Supplementary Table 2. The expression values were normalized with the results of mean values of *eF1* the using 2^−ΔΔCt^ method (Livak and Schmittgen, 2001).

### Statistical Analysis

All values are shown as the means of three replicates with standard error (SE). Analysis of variance was performed by Duncan's multiple range test using the SPSS 22.0 program (SPSS 22.0, SPSS Inc., USA). Different significance of means was tested by LSD at *p* < 0.05. Graphs were plotted using Origin 2021 (Origin Lab Corporation, Northampton, MA, United States). The heat map analysis was visualized by TB tools software (Chen et al., 2020).

## Results

### Effects of Supplementary UV-A and FR Lights on Plant Biomass and Morphological Properties

**Figure 2:**
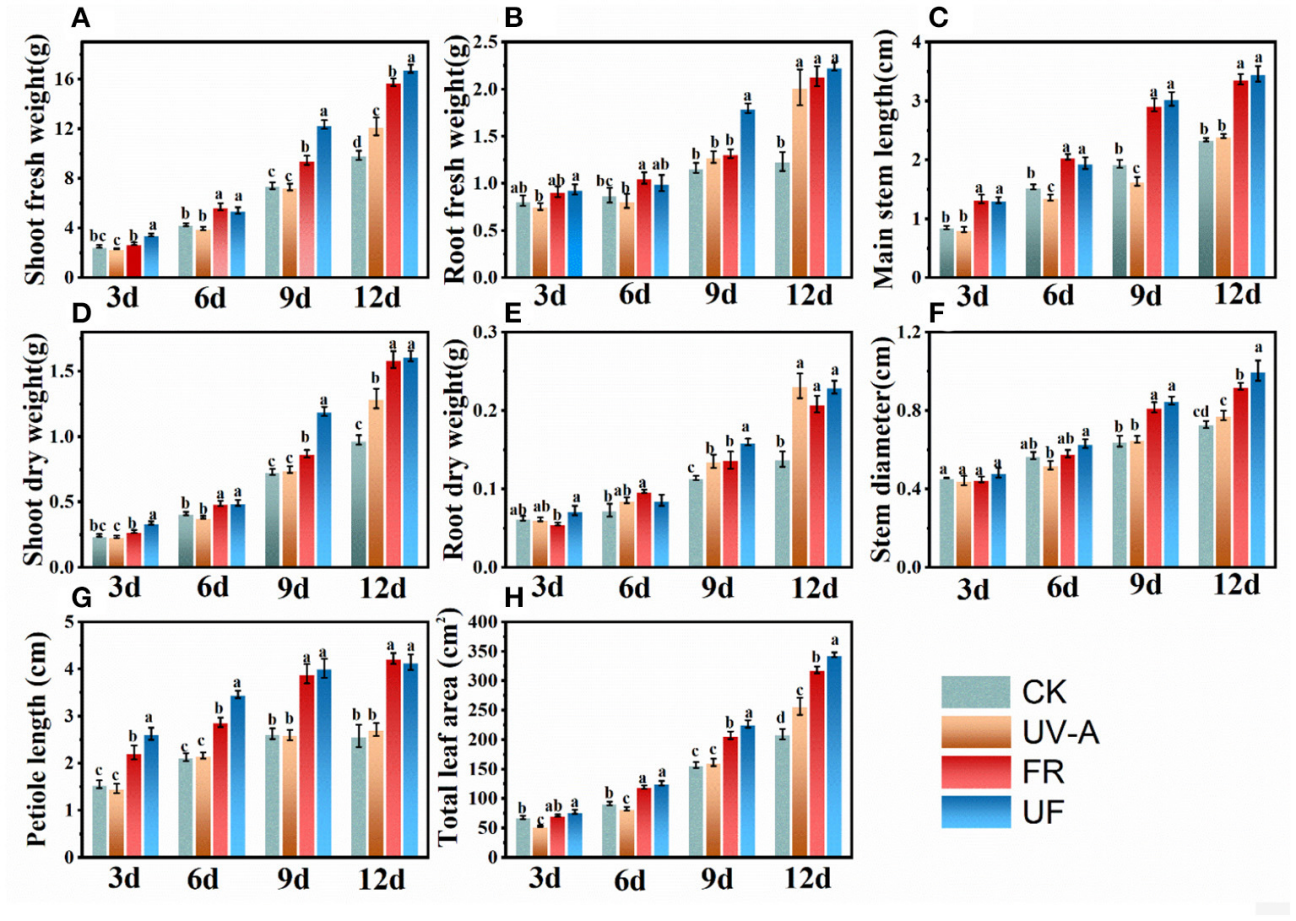
Biometric parameters of Chinese kale baby leaves under different supplementary lightings. Fresh weight of **(A)** shoot and **(B)** root. **(C)** Main stem length. Dry weight of **(D)** shoot and **(E)** root. **(G)** Petiole length of the third leaf. **(H)** Total leaf area. Different letters on the top of the columns mean significant differences at *p* < 0.05 according to Ducan's multiple range test.

The effects of UV-A and FR radiation on the biometric parameters in Chinese kale baby leaves are presented in Figure 2. UV-A slightly inhibited the growth in Chinese kale baby leaves until 6 days, resulting lower fresh and dry weight and the leaf area. No significant differences of these growth parameters were observed between UV-A and CK at 9 days, while obvious growth promotion effects were found at 12 days. The plants under FR and UF exhibited higher fresh and dry weight than CK from 3 to 12 days (Figures 2A,B,D,E), with a pronounced increase in main stem length (Figure 2C), stem diameter (Figure 2F), petiole length (Figure 2G), and leaf area (Figure 2H).

with a pronounced increase in petiole length, stem diameter, main stem length, and leaf area. Therefore, effects of supplemental UV-A lighting on Chinese kale baby leaves growth depended on the growth stage of plants, where UV-A presented a negative effect on biomass at the early vegetative stage while enhanced the yield at the later stage. UV-A and FR exerted a statistically positive effect on the increase of petiole and main stem length and leaf area, and higher biomass was obtained in the combined UV-A + FR treatment (UF) than under UV-A or FR treatments.

### Effects of Supplementary UV-A and FR Lights on the Antioxidant Capacity and Antioxidant Compound Contents of Chinese Kale Baby Leaves

**Figure 3:**
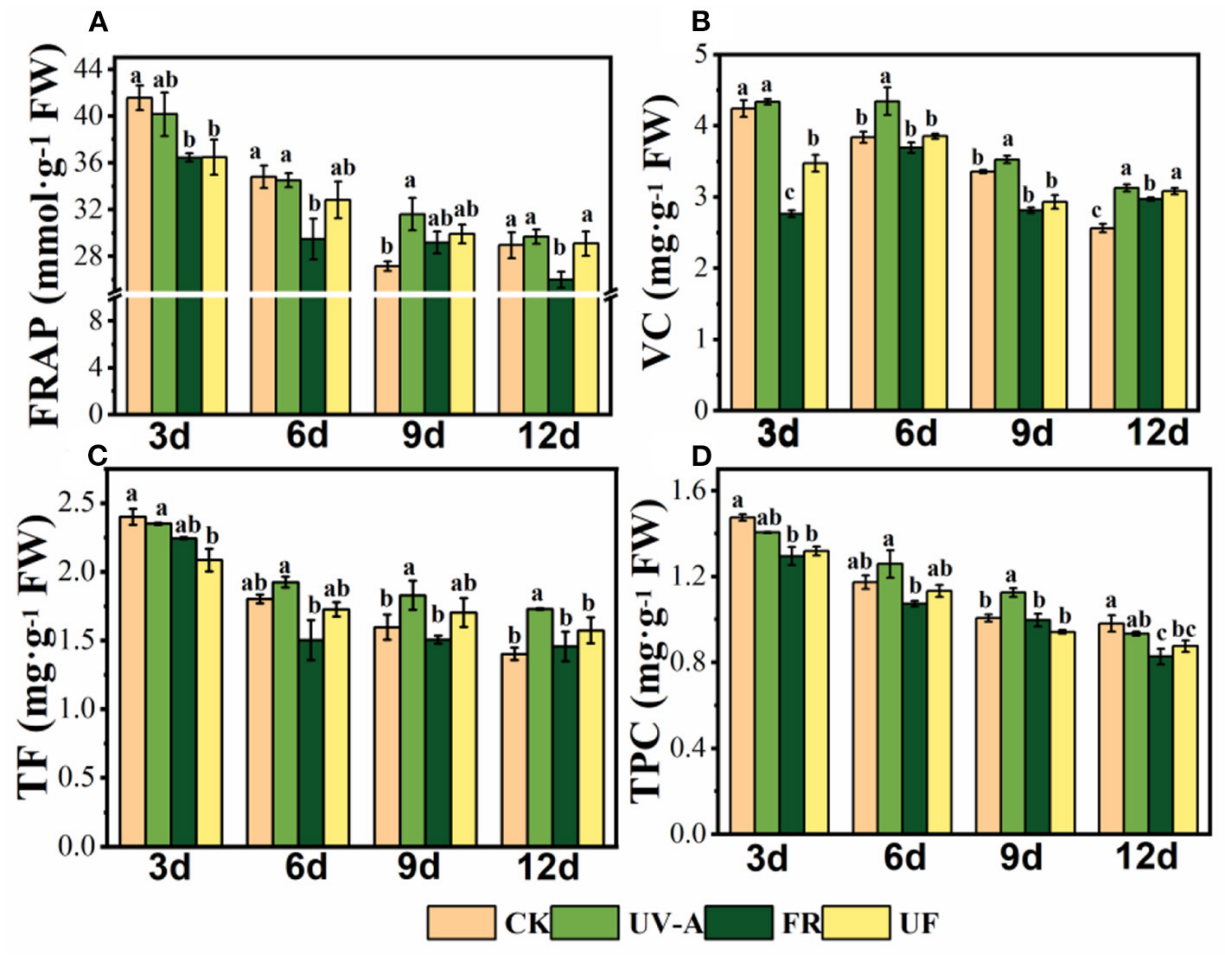
Antioxidant properties of Chinese kale baby leaves under different supplementary lightings. **(A)** FRAP, ferric ion reducing antioxidant power, **(B)** VC, Vitamin C, **(C)** TF, total flavonoids, **(D)** TPC, total phenolic compounds. Different letters on the top of the columns mean significant differences at *p* < 0.05 according to Ducan's multiple range test.

The antioxidant capacity and antioxidant compound contents of Chinese kale baby leaves decreased gradually with the plant development (Figure 3). FRAP values of Chinese kale baby leaves showed a significantly reduction under FR at 3, 6, and 12 days, while no significant changes were found under UV-A and UF compared with CK (Except that FRAP value was significantly decreased under UF treatment at 3 days) (Figure 3A). The Vc contents in Chinese kale baby leaves significantly increased under UV-A from 6 to 12 days. Also, obvious enhancement of Vc contents was found in FR and UF at 12 days (Figure 3B). Flavonoids contents under UV-A increased at 6, 9, and 12 days, while no significant changes were found under FR and UF compared with CK. Total polyphenols contents obviously increased under UV-A at 9 days, but no significantly were observed at 3, 6, and 12 days. Moreover, a general trend of a negative effect on polyphenols contents was found in FR and UF compared with CK (Figures 3C,D).

### Effects of Supplementary UV-A and FR Lights on Mineral Elements Contents of Chinese Kale Baby Leaves

There were differences in mineral elements contents in Chinese kale baby leaf shoot tissue at 12 days under supplementary UV-A and FR (Table 1). Contents of N, P, and K exhibited no significant differences among the different lighting supplements except for the K content increased under FR. Opposite effect was observed in Ca contents, with a notable reduction in all supplemental treatments. Similar patterns of changes were found in the Mg and Fe contents, which showed no striking changes in UV-A but a significant decrease in FR and UF. Interestingly, contents of S under UV-A, FR, and UF were prominently higher than under the CK.

**Table 1:** Effects of UV-A and FR on mineral elements contents of Chinese kale baby leaves.

**Treatment**	**N**	**P**	**K**	**Ca**	**Mg**	**Fe**	**S**
	**(g·Kg^−1^DW)**	**(g·Kg^−1^DW)**	**(g·Kg^−1^ DW)**	**(g·Kg^−1^ DW)**	**(g·Kg^−1^ DW)**	**(mg·Kg^−1^ DW)**	**(g·Kg^−1^ DW)**
CK	52.82 ± 0.51a	7.23 ± 0.14a	54.99 ± 0.47b	29.83 ± 0.71a	3.16 ± 0.02a	71.50 ± 4.46a	10.68 ± 0.09c
UV-A	53.45 ± 0.19a	7.02 ± 0.06a	54.08 ± 0.23b	26.33 ± 0.83b	3.05 ± 0.07a	72.53 ± 1.25a	12.57 ± 0.21a
FR	53.20 ± 0.42a	7.23 ± 0.05a	57.56 ± 0.48a	25.17 ± 0.32b	2.75 ± 0.02b	66.17 ± 1.68b	11.48 ± 0.12b
UF	51.91 ± 2.09a	7.06 ± 0.21a	54.59 ± 0.51b	27.01 ± 0.53b	2.79 ± 0.09b	64.24 ± 1.30b	12.01 ± 0.27ab

*Data represent mean ± SE (n = 12). Means followed by different letters were significantly different at p < 0.05, according to Ducan's multiple range test*.

### Effects of Supplementary UV-A and FR Lights on the Glucosinolates Contents of Chinese Kale Baby Leaves

**Figure 4:**
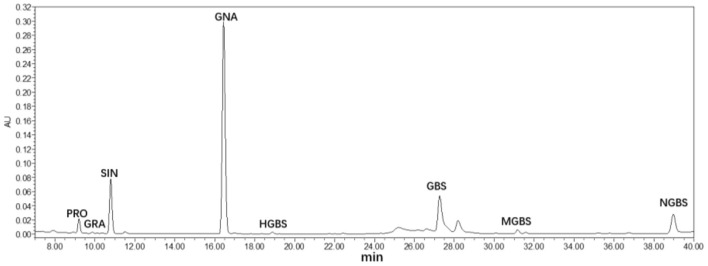
HPLC chromatograms (290 nm) of glucosinolates in Chinese kale baby leaves.

Glucosinolates were extracted and analyzed in Chinese kale baby leaves grown under UV-A and FR supplement from 3 to 12 days (Figure 4). Eight GLs were identified by HPLC in Chinese kale baby leaves (Figure 4), which comprised four aliphatic GLs [progoitrin (PRO), glucoraphanin (GRA), sinigrin (SIN), glucobrassicanapin (GNA)], and four indole GLs [4-hydroxy-glucobrassicin (HGBS), glucobrassicin (GBS), 4-methoxy-glucobrassicin (MGBS), and neoglucobrassicin (NGBS)].

**Figure 5:**
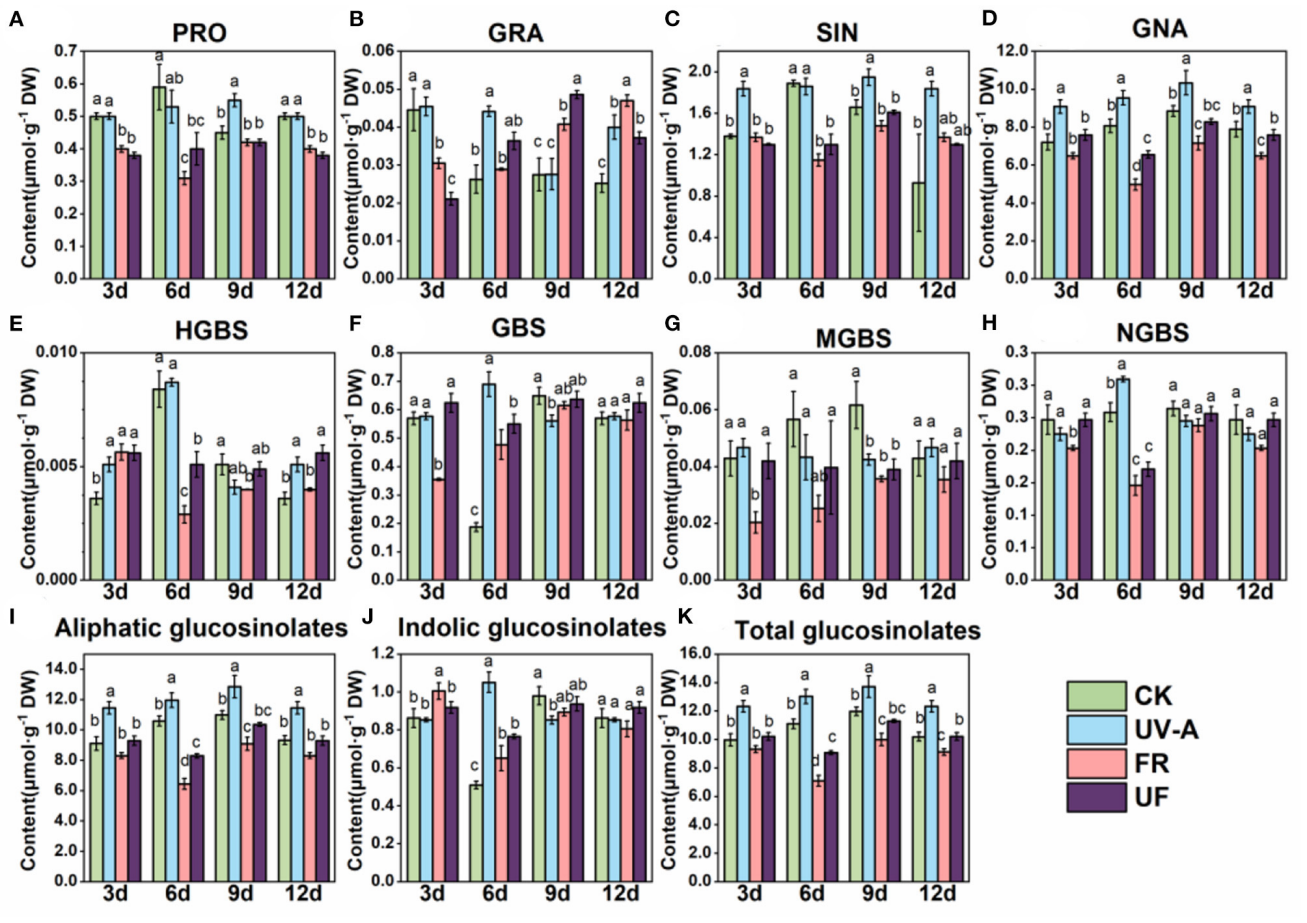
The effect of supplemental UV-A and FR on glucosinolates composition and contents in shoots of Chinese kale baby leaves. The contents of **(A)** PRO, Progoitrin; **(B)** GRA, glucoraphanin; **(C)** SIN, sinigrin; **(D)** GNA, glucobrassicanapin; **(E)** HGBS, 4-hydroxy-glucobrassicin; **(F)** GBS, glucobrassicin; **(G)** MGBS, 4-methoxy-glucobrassicin; **(H)** NGBS, neoglucobrassicin; **(I)** individual aliphatic glucosinolates; **(J)** individual indolicglucosinolates; **(K)** total glucosinolates. Different letters on the top of the columns mean significant differences at *p* < 0.05 according to Ducan's multiple range test.

There was no significant difference in glucosinolates accumulation in different growth stages (Figure 5). GNA and SIN were the predominant GLs in Chinese kale baby leaves, accounting for 65 and 15% of the total GLs content, respectively (Figure 5). The largest increase of total and aliphatic GLs was observed in plants exposed to UV-A (Figure 5), mainly contributed to the enhancement of GNA and SIN accumulation. However, FR had opposite effects that decreased the contents of total and aliphatic GLs, while no striking different or a reduction trend was found in UF.

### Heatmap Analysis of Growth and Nutritional Aspects of Chinese Kale Baby Leaves Under Supplemental UV-A and FR Light

**Figure 6:**
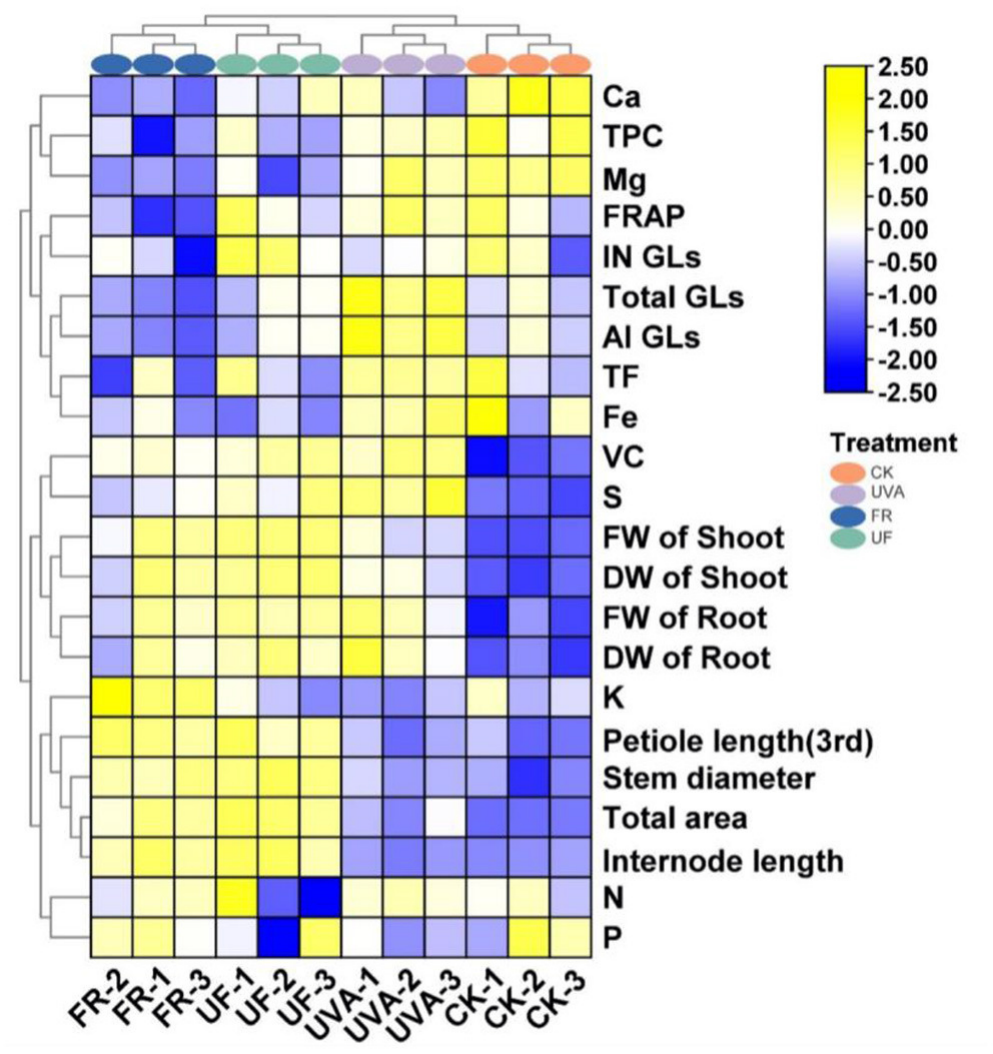
Heatmap analysis summarized the effect of different supplemental UV-A intensities on growth and quality of Chinese kale baby leaves. Results were visualized using a false color scale, with yellow and blue indicating an increase and a decrease.

To gain an integrated view on agronomic traits, a nutritional and functional profile of Chinese kale baby leaves in response to UV-A and FR radiation, the heatmap synthesized was performed (Figure 6). The results demonstrated that four treatments could be divided into two clusters; the first group contained CK and UV-A, characterized by higher contents of glucosinolates, flavonoids, polyphenols, while lower growth-related parameters, especially under CK. FR, and UF displayed higher plant dry and fresh weight, leaf area, petiole length, and main stem length than CK and UV-A. Thus, UV-A was benefited for improving the functional components of Chinese kale baby leaves, while UF and FR were propitious to growth of Chinese kale baby leaves.

### Effects of Supplementary UV-A and FR Lights on Light-Signaling Components of Chinese Kale Baby Leaves

**Figure 7:**
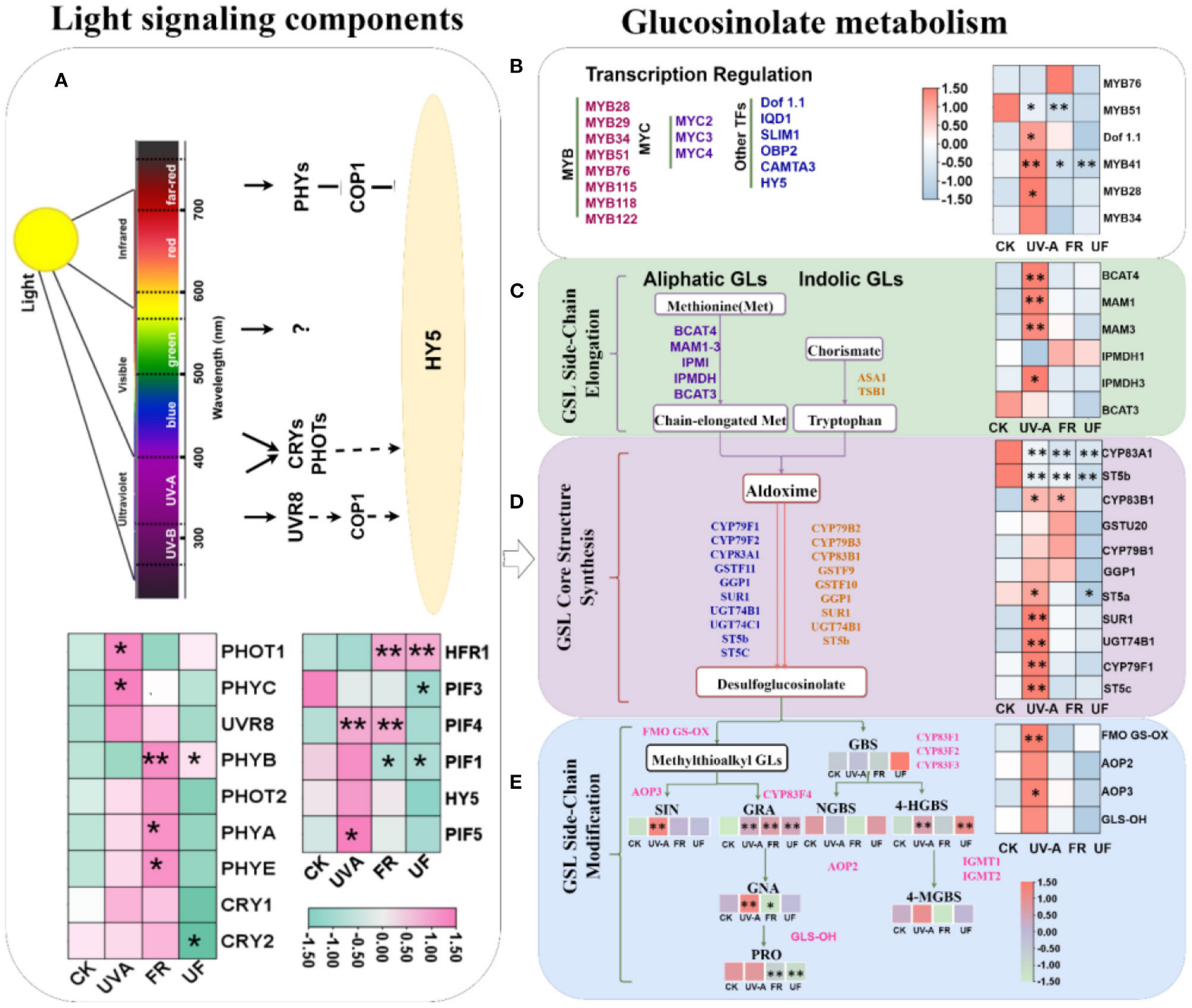
The expression of related genes involved in glucosinolate biosynthesis in 6-day Chinese kale baby leaves grown under supplementary UV-A and FR. Five biosynthetic processes including **(A)** light signaling components; **(B)** the transcription factors involved in GLs biosynthesis; **(C)** chain elongation, **(D)** core structure formation, and **(E)** side-chain secondary modification were illustrated. ^*^, ^**^ represent significant at *p* < 0.05, *p* < 0.01, *p* < 0.001, respectively.

To gain further insights into what changes induced by UV-A and FR radiation in light-signaling pathways, the expressions of genes encoding PHYs, CRYs, and UVR8 and PIFs in Chinese kale baby leaves at 6 days were analyzed. Interestingly, similar gene expression patterns were observed for *CRY1, PHOT1*, and *PHYC*, where the expression did not differ significantly in FR and UF, but markedly higher transcript levels in UV-A than CK (Figure 7; Supplementary Figure 2). FR elevated the expression level of *CRY2* and *PHOT2*, but UF decreased the expression level of *CYR2, PHOT2*, and *PHYE*. Both FR and UF significantly elevated the expression level of *PHYB*. Moreover, *PHYA* displayed a higher expression level in FR than other treatments. However, the expressions of *UVR8* did not show any difference in all treatments. These results implied that UV-A and FR regulated Chinese kale baby leaves growth by some special photoreceptors.

The expression of *HY5* revealed a slight upregulation in UV-A, while decreased in UF. FR and UF lead to a marked upregulation in *HFR1*, while inhibited the expression of *PIF1* (Figure 7; Supplementary Figure 3). *PIF3* expressions decreased in all treatments, while *PIF5* expression under UV-A observably exceeds other treatments (Figure 7; Supplementary Figure 3). Compared with CK, the expression of *PIF4* was significantly upregulated under UV-A and FR, but no significant difference between CK and UF. The expression level of *PIF5* significantly increased under UV-A; the lower expression level was found under FR and UF than CK (Figure 7; Supplementary Figure 3).

### Effects of Supplementary UV-A and FR Lights on the Transcript Levels of Genes Related to Glucosinolate Compound of Chinese Kale Baby Leaves

The expressions of transcription factors and key genes involved in biosynthesis of glucosinolates in Chinese kale baby leaves were analyzed (Figure 7; Supplementary Figure 4). Similar gene expression patterns were observed for *DOF1.1, MYB41, MYB28*, and *MYB34*, where the expression levels were higher in Chinese kale baby leaves grown under UV-A than CK. Except that *MYB76* expression was significantly upregulated under FR treatment, the expressions of *DOF1.1, MYB41, MYB51, MYB28*, and *MYB34* were no significantly different or significantly downregulated under FR and UF.

The upregulated expressions by UV-A radiation were found in the majority of GLs biosynthesis-related genes in the chain elongation *(BCAT4, IPMDH3, MAM1*, and *MAM3*), core structure formation (*CYP79F1, CYP83B1, GGP1, ST5a, ST5c, SUR1*, and *UGT74*B1), as well as side chain modification (*AOP2, AOP3, FMOGS-OX5*, and *GLS-OH*) (Figure 7; Supplementary Figures 5, 6, 7). However, downregulated or unchanged expressions of GLs biosynthesis-related genes in Chinese kale baby leaves were observed in FR and UF.

## Discussion

### Effects of Supplementary UV-A and FR Light on the Plant Morphology and Biomass of Chinese Kale Baby Leaves

Light is an essential external environmental factor playing decisive roles in regulating plant growth and development (Wong et al., 2020). Supplementary UV-A has been used as an efficient light source to meet the demand for higher health-promoting phytonutrients accumulating in horticultural products (Matsuura et al., 2013). Additionally, FR played predominant roles for the elongation of plant internodes and the increase of plant height and biomass (Park and Runkle, 2018; Zou et al., 2019). In the present study, UV-A exerted a suppressing effect on petiole length and the leaf area of Chinese kale baby leaves from 3 to 6 days (Figure 2). Interestingly, with the inhibitory effect gradually disappearing, UV-A was effective in stimulating overall plant growth at 12 days, resulting in a significant increase in growth-related parameters (Figures 2, 5). The promoting effects were consistent with other results that UV-A was positively associated with biomass production in many horticultural and agricultural crops (Chen et al., 2019; Lee et al., 2019). FR significantly promoted the elongation of plant stems and expansion of leaves due to the increasing of cells size, which was induced by FR radiation (De Wit et al., 2016). Thereby FR supplementation was benefit for plant to facilitate light interception and ultimately elicit better biomass production (Possart et al., 2014; Sheerin and Hiltbrunner, 2017). Morphology of Chinese kale baby leaves significantly affected by FR and UF from the third day of treatment, with the leaf area, main stem length, petiole length, as well as the fresh and dry weight of the plant was strikingly increasing (Figure 2). From cluster analysis (Figure 6), it directly indicated that application of UV-A and FR for vegetables might represent a valuable strategy to increase plant biomass.

The morphology and growth of plants responded to different light qualities *via* specific photoreceptors, which were closely related to downstream signaling transduction elements. CRYs, PHOTs, and UVR8 act as UV-A photoreceptors, which were involved in many plants physiological processes, such as stomatal opening, tropism, and hypocotyl elongation response. In this study, the expression of *CRY1* and *PHOTO1* in Chinese kale baby leaves under UV-A was higher than those of CK, while the expression of *CRY2* and *PHOTO2* did not show similar patterns (Figure 7; Supplementary Figure 2). Phytochromes, a group of receptors sensitive to FR light, have vital impacts on shoot and petiole elongation (Holopainen et al., 2018). *PHYA* and *PHYB* expressions were significantly upregulated under FR (Figure 7; Supplementary Figure 2). The promotion effects on plant growth and development under FR and UF might be attributed to the role of phytochromes in regulating the accumulation of various growth-promoting hormones, such as auxin, gibberellin, cytokinin, and brassinolide (Tao et al., 2008). The mechanistic understanding of how plants integrate complex photoreceptor and signaling network from different light environment needs to be investigated further.

Light-activated photoreceptors could move into the nucleus, where they interact with transcription factors, such as *HY5, PIFs*, and *HFR1*, ultimately regulating various light responses. PIF3 and PIF4 could mediate plant cell elongation and growth by positively regulating auxin biosynthesis and signaling (Al-Sady et al., 2006). Meanwhile, *PIF4* takes part in signaling of the R/FR ratio in SAS response. In this study, *PIF3* and *PIF4* in Chinese kale baby leaves showed a significant upregulation under UF and FR, respectively (Figure 7; Supplementary Figure 3). Low-ratio R/FR light irradiation might reduce the activated state of PHYB; once the PIFs dissociated from the PHYB-PIF system, it combined with auxin synthesis-related genes to enhance the elongation of stems, petioles, and leaves (De Lucas et al., 2008; Feng et al., 2008). Higher expressions of *HFR1* were significantly observed in FR and UF (Figure 7; Supplementary Figure 3). Interestingly, HFR1 could interact with PIF4 and PIF5, forming non-functional heterodimers that prevent PIFs binding to target genes by heterodimerization, therefore avoiding exaggerated shade avoiders (Tavridou et al., 2020).

It was worthwhile to note that lower photosynthetic pigments were induced by FR irradiation in several studies, whereas the photosynthetic rate increased, which might be contributed to the Emerson effect (Emerson et al., 1957; Bae et al., 2017). A previous study reported that chlorophyll contents in lettuce leaves increased under UV-A supplementation, while a downward trend was found in FR and UF, and the lowest chlorophyll content was under FR (He et al., 2021). A similar trend was also exhibited in the Chinese kale baby leaves (Supplementary Figure 8). The reduction of chlorophyll content in the Chinese kale baby leaves might be attributed to decreasing of Ca, Mg, and Fe contents under FR and UF treatments (Table 1), which play important roles in chlorophyll synthesis and photosynthesis in plants. Moreover, UF led to overall higher biomass production compared to other treatments (Figure 2), which was consisted with previous study that the combination of UV-A and FR exhibited a remarkable increase in the biomass of lettuce (He et al., 2021). These were consisted with that a combination of UV-A and FR exhibited a remarkable increase in the biomass of lettuce (He et al., 2021). These results indicated that UV-A and FR radiations exerted interactive and superimposing impacts on promoting Chinese kale baby leaves plants growth.

### Effects of Supplementary UV-A and FR Light on Healthy Functional Compound Contents of Chinese Kale Baby Leaves

FRAP values and the contents of total phenols and flavonoids are usually used to evaluate total antioxidant capacity of vegetables. The linear relationship between the total antioxidant activity and the total phenolic content of Chinese kale baby leaves under supplementary UV-A and FR at 12 days is shown (Supplementary Figure 9). *R*^2^ values from these correlations were high (*R*^2^ > 0.9); it was concluded that phenolic compounds were responsible for the antioxidant activity (Jacobo-Velázquez and Cisneros-Zevallos, 2009). Higher slopes were observed in UV-A and UF, which indicated that higher total phenolic content and total antioxidant activity in these treatments than CK and FR. Previous study showed that the FRAP value and contents of flavonoids and phenolics in red- and green-leaf pak-choi under supplementary UV-A (380 nm) were significantly higher than that under CK (Mao et al., 2020). However, FR and UF led to a reduction in levels of DPPH and FRAP and the content of anthocyanins, weakening antioxidant capacity of lettuce (He et al., 2021). In this study, antioxidants contents (total phenols, total flavonoids, and Vc) and antioxidant capacity (FRAP) in Chinese kale baby leaves were generally improved by UV-A, while a decreasing trend was detected in FR (Figure 3). Compared with no light supplementation treatment, a 3.7 W·m^−2^ UV-A fluorescent lamp (λ_max_ = 352 nm) supplemented for 3 days promoted the accumulation of total polyphenols in red leaf lettuce, increasing total antioxidant capacity (Lee et al., 2014). Moreover, UV-A upregulated the expression of transcription factor *MYB*, key genes related to the phenylpropane metabolic pathway (e.g., *PAL, CHS*) by increasing the *CRY1* and *HYH* transcription in plants, and ultimately promoted the accumulation of phenolic and flavonoids in the plants (Fuglevand et al., 1996; Guo and Wang, 2010; Zhang et al., 2013). The reduction in plant antioxidant capacity and antioxidant contents under FR radiation might be due to rapid increase in biomass of Chinese kale baby leaves, which led to the dilution of phytochemicals. Additionally, plants usually balance resource allocation between competing physiological activities (Huot et al., 2014). Plants under FR illumination dedicated most of the available resources to extend growth, reduce branching, and decrease production of some biochemical compounds, such as flavonoids and polyphenols. Therefore, substituting UV-A radiation, which was well-known as an available resource to enhance the accumulation of secondary metabolites and tailor functional features, could offset the negative effects induced by far-red light.

### Supplementary UV-A and FR Influenced the Glucosinolates Accumulation by Affecting Genes Involved in Glucosinolates Biosynthesis of Chinese Kale Baby Leaves

Glucosinolates are a class of secondary metabolites, which is effective in chemoprevention of cancers and degenerative diseases (Johnson, 2002). Light quality plays a vital role in the regulation of glucosinolates metabolism pathway in *Brassicaceae* vegetables. GNA and SIN, the predominant GLs in Chinese kale baby leaves, dramatically increased by UV-A, attributing to an obvious increase in total aliphatic GLs and total GLs contents. Whereas, the accumulation GLs was restrained by FR (Figure 4), which was consisted with previous study that Chinese kale exposure under FR light showing a significant reduction inGLs profiles (Li et al., 2021). Interestingly, GLs contents under UF were higher than under FR but lower than under UV-A, although no obvious difference was found between UF and CK (Figure 4). The accumulations of 3-indolylmethyl glucosinolate, 4-methoxy-3-indolylmethyl glucosinolate, and 1-methoxy-3-indolylmethyl glucosinolate in Brussels sprouts were significantly enhanced by additional UV-A radiation (365 nm) (Rechner et al., 2017). Similarly, 259 kJm^−2^·d^−1^ UV-A radiation was more effective than blue light in increasing the content of 3-methylindole glucosinolate in cabbage (Acharya et al., 2016). Supplementary UV-A to FR was efficient to compensate the reduction in GLs contents induced by FR (Figure 4). Therefore, the combined treatment contributed to higher antioxidant activity in UF compared to FR.

The biosynthesis of GLs can be divided into independent steps: (I) side-chain elongation of selected precursor amino acid, (II) formation of the core glucosinolates structure, and (III) secondary modifications of the side chain of core glucosinolates (Harun et al., 2020). Transcription factors act as important components of the regulatory network controlling glucosinolate biosynthesis. Overexpression of *AtMYB28, AtMYB29*, and *AtMYB76* in Arabidopsis resulted in the production of large amounts of GLs, whereas *AtMYB34, AtMYB51*, and *AtMYB122* specifically regulate indolic GLs formation (Celenza et al., 2005; Gigolashvili et al., 2007). Besides, *IQD1* and *AtDof.1* also played vital roles in the regulation of GLs biosynthesis (Gigolashvili et al., 2008). In this study, *DOF1.1, MYB41, MYB28*, and *MYB34* expression levels were higher in Chinese kale baby leaves grown under UV-A, while a decreasing or unchanged tendency was found under FR and UF. These results indicated that UV-A and FR impacted the accumulation of GLs in Chinese kale baby leaves by affecting the transcription levels of transcription factors related to GLs synthesis.

The glucosinolate biosynthetic pathway involves multiple gene families, such as BCAT, MAM, CYP79, CYP83, and AOPs (Harun et al., 2020). UV-A upregulated expressions of genes related to glucosinolate side chain extension (*BCAT4, IPMDH3, MAM1*, and *MAM3*), core structural (*CYP79F1, CYP83B1, ST5a, ST5c, SUR1*, and *UTG74B1*), and secondary R side chain modification (*AOP2, AOP3, FMOGS-OX5*, and *GLS-OH*) (Figure 7), while no significant or opposite changes in these gene expression patterns of selected glucosinolate-related genes were observed in FR and UF (Figure 7). These revealed that key enzymes functioning upstream of desufo GLs were downregulated by supplementary FR, which might be correlated with the downregulation of the transcriptional activators *MYB28, MYB28-like*, and *MYB51* (Li et al., 2021). Low R: FR ratios (0.55) effectively suppressed the accumulations of indolyl-3-methyl glucosinolate and total indole GLs in Arabidopsis *via* inhibiting key genes involved in indole GLs biosynthesis, such as *CYP79B2, CYP79B3*, and *CYP83B1*/*SUR2* (Celenza et al., 2005). In addition, our results also showed that sulfur contents were increased by all supplemental lights (Table 1), which indicated that sulfur might participate in GLs biosynthesis induced by light. Moreover, the GLs accumulation in plants does not only depend on their biosynthesis and the transport of GLs between organs, and the degradation of GLs in tissues also has a greater impact on the final total GLs content (Velasco et al., 2007; Wu et al., 2017). Therefore, alone or interactive effects of UV-A and FR on modulating GLs metabolism need to be further explored.

## Conclusions

The morphological, physiological, and biochemical characteristics of Chinese kale baby leaves were altered by UV-A, FR, and UF radiation. FR light had a more positive impact than UV-A on the Chinese kale baby leaves growth, with a greater growth-related parameter, while it resulted in a reduction in the phytochemicals. UV-A displayed a remarkable effect on the contents of Vc, total phenolic, and flavonoid, as well as total aliphatic glucosinolates and total glucosinolates. Moreover, transcription factors (*DOF1.1, MYB41, MYB28*, and *MYB34*) and genes related to cure structure of glucosinolates (*BCATs, MAMs, CYP79s, CYP83s*, and *AOPs*) and other gene families were upregulated under UV-A, which contributed to higher GLs accumulation in Chinese kale baby leaves. However, downregulated or unchanged expressions of GLs biosynthesis-related genes in Chinese kale baby leaves were observed in FR and UF. Therefore, UV-A was benefited for the production of functional vegetables, while FR was conducive to a significant increase in crop yield. Supplemented with UV-A to FR seemed to be efficient to compensate the decrease in phytochemicals such as GLs contents induced by FR. Thus, the combination of UV-A and FR, as a balance between biomass production and the production of secondary metabolite, provided a new perspective for the application of artificial light in horticultural crop production.

## Data Availability Statement

The datasets presented in this study can be found in online repositories. The names of the repository/repositories and accession number(s) can be found in the article/Supplementary Material.

## Author Contributions

RH conceived, designed, and performed the experiments and wrote the manuscript. YL, SO, and MG performed the experiments and statistical analysis. YZ and SS were involved in the supervision of the experiments and analyzed the results. HL conceived and designed the experiments, analyzed the results, contributed to manuscript revision, and read and approved the submitted version. All the authors have read and approved the manuscript for publication.

## Funding

This work was supported by a grant from the Key-Area Research and Development Program of Guangdong Province (2019B020214005 and 2019B020222003).

## Conflict of Interest

The authors declare that the research was conducted in the absence of any commercial or financial relationships that could be construed as a potential conflict of interest.

## Publisher's Note

All claims expressed in this article are solely those of the authors and do not necessarily represent those of their affiliated organizations, or those of the publisher, the editors and the reviewers. Any product that may be evaluated in this article, or claim that may be made by its manufacturer, is not guaranteed or endorsed by the publisher.
