# Supplementary material for: Regulation of Growth and Main Health-Promoting Compounds of Chinese Kale Baby-Leaf by UV-A and FR Light
Source: Front Plant Sci. 2021 Dec 17;12:799376. doi: 10.3389/fpls.2021.799376 (PMC8719463; doi:10.3389/fpls.2021.799376)
Supplement: Supplementary file 1 [file Data_Sheet_1.docx]

Supplementary Material

# Supplementary Figures and Tables

##
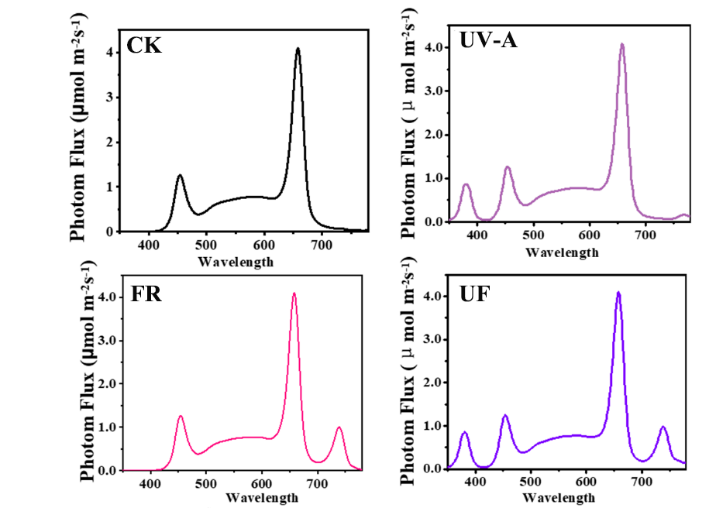
Supplementary Figures

**Supplementary Figure 1**. Photon flux density in the five treatments.





**Supplementary Figure 2.** Expressions of photoreceptors genes in Chinese kale baby-leaves supplemented by UV-A and FR. Vertical bars are means ± SE (n = 3). Bar labeled with lowercase letters are significantly different by one-way ANOVA with Duncan’s multiple range test (p ≤ 0.05).





**Supplementary Figure 3.** Expressions of transcription factors genes involved in light signal transduction in Chinese kale baby-leaves supplemented by UV-A and FR. Vertical bars are means ± SE (n = 3). Bar labeled with lowercase letters are significantly different by one-way ANOVA with Duncan’s multiple range test (p ≤ 0.05)

**Supplementary Figure 4.** Expressions of transcription factors genes involved in glucosinolate biosynthesis in Chinese kale baby-leaves supplemented by UV-A and FR. Vertical bars are means ± SE (n = 3). Bar labeled with lowercase letters are significantly different by one-way ANOVA with Duncan’s multiple range test (p ≤ 0.05)






**Supplementary Figure 5.** Expressions of glucosinolate side chain extension genes in Chinese kale baby-leaves supplemented by UV-A and FR. Vertical bars are means ± SE (n = 3). Bar labeled with lowercase letters are significantly different by one-way ANOVA with Duncan’s multiple range test (p ≤ 0.05)

**Supplementary Figure 6.** Expressions of glucosinolates core structure formation genes in Chinese kale baby-leaves supplemented by UV-A and FR. Vertical bars are means ± SE (n = 3). Bar labeled with lowercase letters are significantly different by one-way ANOVA with Duncan’s multiple range test (p ≤ 0.05)





**Supplementary Figure 7.** Expressions of glucosinolates side-chain secondary modification genes in Chinese kale baby-leaves supplemented by UV-A and FR. Vertical bars are means ± SE (n = 3). Bar labeled with lowercase letters are significantly different by one-way ANOVA with Duncan’s multiple range test (p ≤ 0.05)





**Supplementary Figure 8.** Pigments contents in Chinese kale baby-leaves supplemented by UV-A and FR. Vertical bars are means ± SE (n = 3). Bar labeled with lowercase letters are significantly different by one-way ANOVA with Duncan’s multiple range test (p ≤ 0.05).

**Supplementary Figure 9.** Correlation between total phenolics and total antioxidant activity under different supplementary lightings. The antioxidant activity was measured by either **(A)** 2,2-diphenyl-1-picrylhydrazyl (DPPH) or **(B)** ferric-reducing antioxidant power (FRAP) methods.


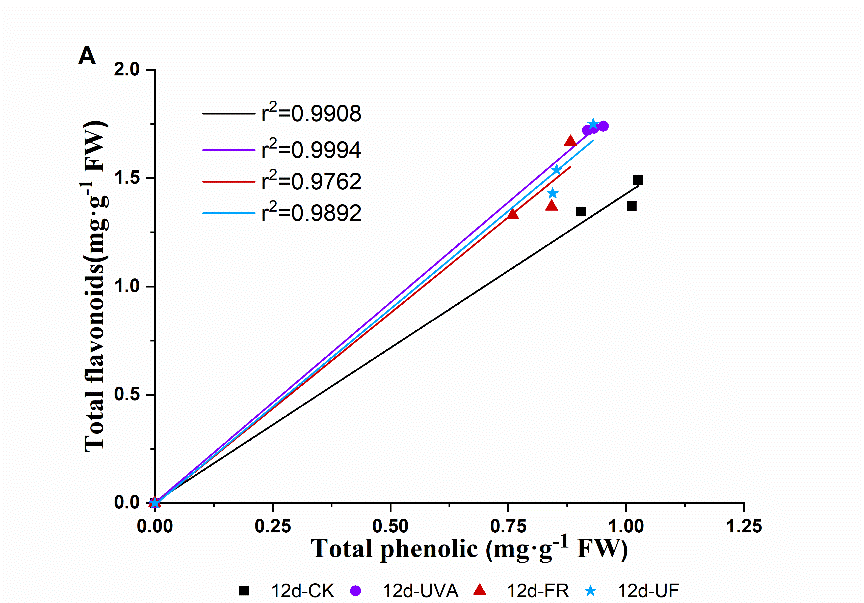

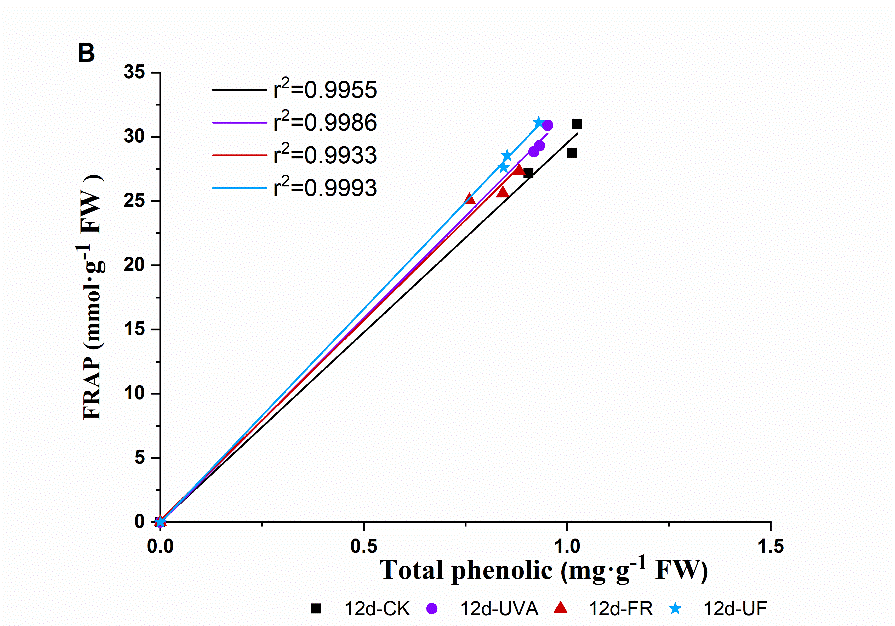


## Supplementary Tables

**Supplementary Table 1.** The concentration of mineral element in this study.

| Mineral element | Concentration (mg·L^−1^) | Mineral element | Concentration (mg·L^−1^) | |
| --- | --- | --- | --- | --- |
| N | 210 | Fe | 5.6 |  |
| P | 31 | B | 0.5 |  |
| K | 234 | Mn | 0.5 |  |
| Ca | 160 | Zn | 0.05 |  |
| Mg | 48 | Cu | 0.02 |  |
| S | 64 | Mo | 0.01 |  |

# Supplementary Table 2. Primers used for qRT-PCR analysis.

| **Gene name** | **Primer sequence (5'→3')** | **Length (bp)** | | |  |  |
| --- | --- | --- | --- | --- | --- | --- |
| *CRY1* | F: CAGCAGCAGGAGGCAAGA | | | 18 | |  |
|  | R: CTGACCACTCGGGGACAA | | | 18 | |  |
| *CRY2* | F: AGCAGCGCCTGATGAGAT | | | 18 | |  |
|  | R: CAGAGGGGCAAAGAAGACC | | | 18 | |  |
| *PHOT1* | F: TGCTTAACCGTAACAAAGTGC | | | 21 | |  |
|  | R: CGTAGAGTGCTGGGAGAAAA | | | 20 | |  |
| *PHOT2* | F: TCTACTGAAGTTGAACGAAGCA | | | 22 | |  |
|  | R: CTGGCGTACACTATGGGACA | | | 20 | |  |
| *PHYA* | F: ATGTGGAGTGTCTGCTGTGTGT | | | 22 | |  |
|  | R: GCGGATTCCTTATCTGTCTTTTA | | | 23 | |  |
| *PHYB* | F: F: TCGGAAGTGTCATCAACGC | | | 19 | |  |
|  | R: CTCTTCAGGAATGTCACGGA | | | 20 | |  |
| *HFR1* | F: AGCTCAGGGTATAAATCTGGTG | | | 22 | |  |
|  | R: TGGTAATGCTGTTGTAGGAAAG | | | 22 | |  |
| *HY5* | F: AAAATGCAACCTCCACCGCT | | | 20 | |  |
|  | TCCTCTCCCTCGCTTGTTGA | | | 20 | |  |
| *PIF1* | F: AAACTCGTATGGGCGGCTAC | | | 20 | |  |
|  | R: GCGGAATTGCTGAAATGGA | | | 19 | |  |
| *PIF3* | F: GTGGTTTCTTCACGGGTTCT | | | 20 | |  |
|  | R: CTGTCGTCGTTTCATCTTTTGT | | | 22 | |  |
| *PIF4* | F: TGGATGGGGAATGGAATGGC | | | 20 | |  |
|  | R: GTCCCGCCTGAGAACCAAAT | | | 20 | |  |
| *PIF5* | F: CAAATGTTGTCGGAGCAGC | | | 19 | |  |
|  | R: CATACGAAGACTGTCGGTGGT | | | 21 | |  |
| *Dof1.1* | F: AAGGCTTGCCGTCGTTACTGG | | | 21 | |  |
|  | R: ACTTGGAGCGTTGACCGTGGA | | | 21 | |  |
| *MYB41* | F: GCTCGGCTTGTCTCCTGA | | | 18 | |  |
|  | R: CTCCATAAAACGCCTAACTTCT | | | 22 | |  |
| *MYB51* | F: TGAACAAGGTAGCAAATAGGG | | | 21 | |  |
|  | R: ACGAAGTTGGTGTGAAGGAA | | | 20 | |  |
| *MYB76* | F: TCTTCTTGGAAACAGGTGGG | | | 20 | |  |
|  | R: CGATGCCGCTTCTAACGA | | | 21 | |  |
| *MYB28* | F: CTCTAATCGCTCTACATATCATCC | | | 24 | |  |
|  | R: CCCTTTCTTCAAACCCATCT | | | 20 | |  |
| *MYB34* | F: TATCTAAGACCCGACATCAAGAG | | | 23 | |  |
|  | R: GTCGTTGTCAGTTCGTCCC | | | 19 | |  |
| *BCAT3* | F: CCTTCACTCGGAACTTATCGC | | | 21 | |  |
|  | R: TGTCCCAATCTATGTCGGCTA | | | 21 | |  |
| *BCAT4* | F: TCCAAGAGGGGAAGATCGTT | | | 20 | |  |
|  | R: GTCTGGTCGGAATAGCACAA | | | 20 | |  |
| *IPMDH1* | F: GATGGCTCTGTTTTACCTTCG | | | 21 | |  |
|  | R: TCTTGATTCCCCTTGGCTCT | | | 20 | |  |
| *IPMDH3* | F: GGATTTGGTCGGAGTGCCCT | | | 20 | |  |
|  | R: CTCATTGTTGTCCCATTTGT | | | 20 | |  |
| *MAM1* | F: TACTCTATCGTTATGGCTTCGTC | | | 23 | |  |
|  | R: CTTGTGCGGTATGTACTCTGG | | | 21 | |  |
| *MAM3* | F: GAATAGGCGAAAGAAGTGGG | | | 20 | |  |
|  | R: TATGGGCTTATGTGGTTGAA | | | 20 | |  |
| *CYP79B2* | F: GAAAATGAGGAAAGTCGTGATG | | | 22 | |  |
|  | R: CCACTTGTTGGCGGAGATA | | | 19 | |  |
| *CYP79F1* | F: ACTATTGAAGCGGACAACCTC | | 21 | | | |
|  | R: TCACAGCGTAACCATAAACCC | | 21 | | | |
| *CYP83A1* | F: GGATGATGGATAAGGTCGGT | | 20 | | | |
|  | R: CGTAGCCAGTAAAAGGGAAA | | 20 | | | |
| *CYP83B1* | F: CCGACCTTTTCCCTTATTTC | | 20 | | | |
|  | R: AAGAGTCTCATCGAGGAGTTCTT | | 23 | | | |
| *GGP1* | F: TAAGAGGAGGGACAGTGGGA | | 20 | | | |
|  | R: CCTGATGTAACTTTAGGATGGC | | 22 | | | |
| *GSTU20* | F: AGGTTTTGGGCTGATTTCGT | | 20 | | | |
|  | R: CTTCACCCCTGCTGCTTGTT | | 20 | | | |
| *ST5a* | F: GTGTTGGGTTACTGGAAGGC | | 20 | | | |
|  | R: TCACAAACGGCAAAGGATTA | | 20 | | | |
| *ST5b* | F: CACGATTTCTTACCCGATTC | | 20 | | | |
|  | R: TTCTCTTGGTGAGCTTTCCA | | 20 | | | |
| *ST5c* | F: AGGGGAGAAAGATAGGGAGG | | 20 | | | |
|  | R: GGTGATGAAGCAAGAAAGCCA | | 20 | | | |
| *SUR1* | F: GAACTGAGAAAGTGCCCAAAA | | 21 | | | |
|  | R: GCAAGGATAGACGGAAGGATC | | 21 | | | |
| *UGT74B1* | F: TTCTTCTTCCTCCTCTTCTGTG | | 22 | | | |
|  | R: GTGGCGATTGTGACTTTGAC | | 20 | | | |
| *AOP2* | F: ATTCACAGTCCCTTGTCGCT | | 20 | | | |
|  | R: TCTGCCTTGTCATAATCCCT | | 20 | | | |
| *AOP3* | F: GAGATTGAAGGTTTGGAGGTG | | 21 | | | |
|  | R: CCAGCCATAACAACGAAAGAGT | | 22 | | | |
| *FMOGS-OX5* | F: TGGATGACATTACTATTCACTCTGTT | | 26 | | | |
|  | R: CGATGCGTGTATCTCTTGGG | | 20 | | | |
